# Predictors and correlates of examination anxiety and depression among high school students taking the Sudanese national board examination in Khartoum state, Sudan: a cross-sectional study

**DOI:** 10.11604/pamj.2019.33.69.17516

**Published:** 2019-05-29

**Authors:** Muwada Bashir Awad Bashir, Israa Mohammed Alfatih Hussein Albadawy, Samuel Nambile Cumber

**Affiliations:** 1 Discipline of Medicine and Surgery, Faculty of Medicine University of Khartoum, Sudan; 2 Section for Epidemiology and Social Medicine, Department of Public Health, Institute of Medicine (EPSO), The Sahlgrenska Academy at University of Gothenburg, Gothenburg, Sweden; 3 Faculty of Health Sciences, University of the Free State, Bloemfontein, South Africa; 4 School of Health Systems and Public Health, Faculty of Health Sciences, University of Pretoria Private Bag X323, Gezina, Pretoria, 0001, Pretoria, South Africa

**Keywords:** Correlation, predictors, exam, anxiety, depression, Sudan

## Abstract

**Introduction:**

Test anxiety and depression are of the major challenges experienced in students' life, considering the inverse associations they have on their mental wellbeing and academic performance. Evidence from Sudan have reported high figures of adolescent's mental health problems of depression and anxiety. However, studies investigating its association with academic exam stress are lacking. We investigated the prevalence of exam anxiety and depression severity among student setting for Sudan national boarding examination, aiming at identifying possible predictors related to student's socio-demographic and academic status and measuring correlation between exam anxiety and depression severity status among students.

**Methods:**

Using cross-sectional design, data obtained using standardized west side anxiety scale for measuring test anxiety; and patient's health questionnaire (PHQ9) of nine items for measuring depression was presented in percentages. Association with sociodemographic and academic factors was measured using logistic regression models. Analysis was run at 0.05 level of significance.

**Results:**

Depression and exam anxiety were found to be highly correlated. The highest fractions of students are those with high levels of test anxiety and moderate to severe depression. Gender, maternal level of education, previous exam experience and academic performance are significant predictor for student's exam anxiety status.

**Conclusion:**

High figures of exam anxiety and depression are there among Sudanese students setting for their third years boarding exam. Males, low academic performance and maternal low education are risk factors. School mental health services and programs addressing such group of students are highly demanded in line with more elaborative research efforts in this arena.

## Introduction

Of late, the psychological impact of the educational process on adolescent's life has become a topic of raising attention. Test anxiety and depression are one of the major challenges experienced by students, considering the inverse associations they have onstudent's mental well-being and academic performance [1-3]. Examinations stress and school work is considered one of the major stress causes in adolescents life,especially when they act as an obstacle in face of their future aspirations [4]. Whether being an interim form of anxiety before critical final exams time or a daily hassles students experience throughout their schooling time, stressors like inefficient study before exam , lack of review of study materials, negative irrational concerns about exam ,study nights and other socio-emotional factors, may causatively or indirectly correlated with other psychological problems like anxiety depression or other forms of diseases and maladjustments [5-7]. Consequences of exam anxiety might affect both students' academic performance or physiological and psychological well-being: students who suffer anxiety could have impaired reasoning abilities, working memories and self-esteem. Furthermore, they might experience psycho-somatic characteristics like palpitation, sweating palms, fast breath, panic attacks and stomach upset [8]. Test anxiety among adolescent students was proven to manifest in associative model with mental and behavioral comorbidities like depression, substance abuse, conduct disorders and intentions to violence and more extremely tendency to commit suicide [9-11].

The educational system in Sudan is graded into four levels: the pre-primary, basic level, secondary level and higher education. The secondary school level is of 3 years, and students age in this level is between 14 to 18 years old. In the third year of the secondary level, students set for the boarding general certification exam which's result determine their future higher educational chances of university admission and their preferred discipline qualifications [12]. In Sudan students' choice and preference for future professions, which are derived by the better employability chances, income and life quality standards are mostly driven towards disciplines like medicine, engineering, law and management. These highly demanding disciplines require very high academic scores to be achieved by students in the national secondary school exams in order to secure a university seat. Such consensus preferences manifest as stress source that put students of the third year in extreme stressful challenges exerted by their own willingness, community and parents and community expectations [13, 14]. Among undergraduate adolescent students in Sudan, test anxiety was found to be significantly higher than the critical value of the test scales, especially among females and students studying certain specializations. Also reported, the significant negative impact test anxiety exerts on students' self-esteem and academic achievements [15]. The estimated prevalence of major depression among Sudanese females aging 12-19 year is 4.2% with 11% of them having severe depression .Such figures increase with age and positively correlate with anxiety findings among these age groups [16, 17]. Studies that specifically investigated exam anxiety and depression correlates among third year high school students in Sudan , despite the considerable presser situation they experience in this stage of their lives, is lacking. Therefore, and in recognition of such facts, this study aims at assessing the level of exam anxiety and depression among high school adolescent students in Khartoum state in Sudan, their impact on students' academic performance and association with their socio-demographic characteristics.

## Methods

Ethical approval for this study was obtained from department of community medicine faculty of medicine, university of Khartoum, high education office in Karrary locality in Khartoum state and the administrative authorities of the participating schools.

**Study area:** this is a cross sectional study that was conducted in Sudan, Khartoum state, Karrary locality in the time duration from March to July 2016. The locality composes of urban and rural set ups. In both, schools are classified into modern, geographical and private school. This classification is based on the maximum accepted academic score for students promoted from the primary level to be admitted in the specific school. Model schools recruit students who have the highest scores in their primary level final exams, while students who scoreless either register in geographic or private schools based on their preferences or financial status. In some parts of the locality, modern schools have further sub classification for students into class A and B classes ,where class A students have higher scores than those in class B classes. To insure the maximum generalizability of our study findings, we included third academic year students from both urban and rural sides of the locality with all types of schools included considering the inclusion of both female and male students.

**Study population:** students in the third year of secondary school who are setting for their final national secondary school board exam were included. Considering the variability in the school entry age in Sudan due to pre-school education conditions and to allow for age variance effect study age range for participants inclusion was from 15-18 years old, considering that the average age of students in the third year is 17 years old [12]. Participants must be going to set for Sudan national secondary school exam of the same year and capable of read and understand the questionnaire used for the data collection in this study.According to information observed from Kararry locality and school's administrative records, the estimated population of students fulfilling the previous criteria is 5280 students. Sampling: using 5% degree of precision an estimated sample size of 377 was determined. Considering lost data due to incomplete questionnaires, an extra 5% of the sample size was collected ending up with total size of 395 students. Multistage cluster sampling technique was used for data collection,through which high schools were classified into geographical clusters and the schools from which participant students will be selected were specified using a random selection method. Furthermore, in the selected school's classes of students were clustered and the participating classes were selected using random sampling.

**Data collection:** data was collected using a self-administered questionnaire consisting of three sections. The first one includes information about student’s social and demographic parameters and academic performance. Students were asked to give the average of their academic performance of the three months prior to the study on a ten units interval scale corresponds to fail, pass, fair, good, very good and excellent. Each interval consists of ten units and the pass limit is 50. The second section of the questionnaire is the west side test scale for state anxiety measure. It is a brief instrument ofanxiety screening aims at identifying state anxiety among students. The scale consists of ten items of which the first six questions test for incapacity or impairment dimension while the following four assess worry and dread ones. It is scored from 1.0 (no anxiety) to 5.0 (panic). A score of 4.0 and above indicates an extreme anxiety status, 3.5 and above: high anxiety status while range of 3.0 to 3.4 signpost a moderate high anxiety status [18]. Despite the high validity and reliability of this scale for anxiety measure, it doesn't include items that assess for the psychological component [15, 19]. So the patient health questionnaire PHQ 9 was used in this study to measure depressive symptoms among students and their psychological statuses and was included in the third section of the questionnaire. The PHQ 9 is a self-administered modification of the PRIME-MD diagnostic instrument of common mental health morbidities that demonstrated a high reliability (Cronbach alpha 0.89), sensitivity (0.897) and specificity (0.989) for measuring depression in studies of similar purpose and set up [20]. The depression module of the questionnaire that is used in this study includes the nine depression criteria being scored from 0 = never at all to 3 = nearly every day. In this study, the module was used for assessing depression severity among students by summating the scale score that is expected to equal a value between 0 and 27 and indicates the depression severity status using the values of 5.10 and 15 as cut off points for minimal, mild and moderate depression, respectively, while a score higher than 15 indicates severe depression [21-23]. For the purpose of analysis in this study, students' status of anxiety and depression were further dichotomized into low and high exam anxiety status and mild and severe depression; value of 3 and above was used as cut off point for anxiety, while 10 and above for depression severity. Twenty students who were excluded from participating in the study fully read and answered the questionnaire and difficulty or ambiguity in the questionnaire items was reported by any. The English version of the questionnaire was used as students' ability to read and understand in English was assured and checked in advance.

**Statistical analysis:** descriptive data was summarized percentages. Students' levels of exam anxiety and depression were dichotomized into high and low exam anxiety and depression statuses. Multivariate backward stepwise logistic regression modeling was used to identify significant predictors of student's level of exam anxiety and depression .Factors of age, gender, parental level of education, students' exam experience and academic performance were preliminary investigated for difference in the mean score of exam anxiety and depression using independent sample t test or ANOVA test as appropriate and those which appear to be of significant difference were later inserted to the model for predication assessment. The model that demonstrated the highest sensitivity and outcome predictivity was selected based on likelihood ratios. Prediction estimates were calculated as odd ratios. Person correlation test was used to assess for correlation between depression and exam anxiety. All the analysis was done at 95% level of confidence using SPSS software version 24.

## Results

***Students socio-demographics and academic performance and exam experience:*** *the obtained response rate was 98% as 388 students fully answered the questionnaire. Out of them, 129(33%) were males while 259(67%) were females. The most frequent age group is between 17 and 15 years old: 357(92%) followed by those of age more than 17 years: 29(8%),and only two students age less than 15 years (1%). Most of the students were studying in the science section 300(77%), in opposite to those studying on the liberal arts academic section who were only 88(23%) students. Paternal level of education was relatively high for most of the participants as father graduated from high school education was reported by 130 (34%), university level: 112(29%) and higher education level: 89 (23%). Monir students' group were of non-educated fathers: 6 (2%) or their fathers only attended khalwa and kutab (a religious form of education based on keeping and learning holy Quran the holy book of Islam through which students acquire only basic reading and writing skills): 24(6%) and 27(7%) of the students reported that their fathers were of basic level of education. Similarly, when it comes to maternal education, most of the students cited that their mothers had high education level with 118 (30%) finished high school education, 112(29%) university level and 67(17%) higher school education. The lesser level of maternal education was presented in 63(16%) of the students who mentioned basic level of education, 16(4%): khalwa and kutab and only 12(3%) with no education. Considering students experience with the exam conditions, most of the students 335(86%) were sitting for Sudan national secondary certificate exam for the first time, while 53(14%) had previous experience of setting for the exam. Out of these, 6(2%) sat for the exam once before, 46(12%) twice before and one student is of three times or more experience. Regarding academic performance, majority of the students' average degrees fell in the very good: 109(28%) and good: 115(30%) classes. Other academic degrees such as excellent: 46(12%), fair: 87(22%), pass: 25(6%) and failure: 6(2%) were of less frequency.*

***Test anxiety predictors:*** *test anxiety assessment revealed that 79(20%) of the students attained the mean score of high normal test anxiety level, 61(16%): moderate high anxiety, 35(9%): high test anxiety and 33(9%) scored the mean of extreme test anxiety status.Overall, students with no anxiety represented 180(35%), while 208(54%) of the students range from high to extreme anxiety. Gender, maternal level of education, previous experience with the exam setting and average academic performance score were the variables significantly predicted student's status of anxiety. females were of lower probability to develop high exam anxiety in comparison to males who had 2.501 times higher odds of having high test anxiety (OR = 2.501, CI:1.894-2.988). Students' previous encounter with the exam setting was also found to significantly predict students' level of exam anxiety (p value = 0.006),as students who set for the exam more than once were more likely to develop high exam anxiety score than students with less exam experience (OR = 4.222, CI:2.643-5.621). Maternal level of education was found to be a negative predictor of student's exam status as university education levels among moms being associated with the lowest exam anxiety status (OR = 0.138,CI:0.082-0.778). In line, low academic performance grades also inversely predicted our outcome; the higher the students reported grades the lower their probability of acquiring high exam anxiety (OR=0.138,CI:0.104-0.269) however other factors of age, paternal level of education and academic section were not predictive of student's exam anxiety status (**Table 1**).*

**Table 1:** Odd ratios of association between student’s exam anxiety and social demographic and academic factors

Variables	category	Logistic coefficients	P value	Odd ratio	95% C.I. for Odds ratio	
Gender	Males	0.917	0.000	2.501	1.894	2.988
Maternal level of education*			0.02			
	Primary education	-1.463	0.013	0.232	0.367	0.707
	Secondary education	-1.171	0.015	0.310	0.342	0.771
	University education	-0.873	0.008	0.418	0.082	0.778
Times of previous experience of exam setting*	More than once	2.170	0.001	4.222	2.643	5.621
Average of academic performance*			0.000			
	pass	-0.412	0.048	0.662	0.191	0.926
	good	-1.323	0.034	0.266	0.341	0.955
	Very good	-1.250	0.010	0.287	0.104	0.471
	Excellent	-1.983	0.036	0.138	0.104	0.269

* (The reference level for maternal level of education is the category non-educated. The reference level for the variable times of previous experience of exam is the category none or one time. The reference level for the variable academic performance level is the category fail. The outcome variable is high exam anxiety)

***Depression severity and predictors:*** *regarding depression severity, 137(35%) of the students had mild depression, 111(29%) had moderate depression, 98(25%) had severe depression and the least squad of the students 42(11%) were of minimal or no depression. Students status of depression was only significantly predicted by their low academic performance. Students who score higher in their assessment exams were of lower probability to develop severe depression in comparison to students who fail their studies (OR=0.287,CI:0.194-0.466). Other factors of gender, age, paternal level of education and exam experience were of no significant predictive value (**Table 2**).*

**Table 2:** Odd ratio of association between students’ depression status and academic performance

	Category	Logistic coefficients	P value	Odds ratio	95% C.I. for odds ratio	
					Lower	Upper
Average of academic performance **
			0.014			
	Good	-0.412	0.030	0.662	0.528	0.745
	Very good	-1.323	0.020	0.266	0.204	0.757
	Excellent	-1.250	0.011	0.287	0.194	0.466

** (The reference level for the variable average of academic performance is category fail. The outcome variable is severe depression )

***Correlation between exam anxiety and depression severity status:*** *depression and exam anxiety were of strong positive correlation indicating that depression severity is possibly due to exam anxiety experienced by the students (Coefficient of correlation = 0.752, p value < 0.000).*

## Discussion

Using a standardized psychological assessment method, this research work investigated exam anxiety and depression levels and their possible predicting variables, among third year board exam taking students in secondary school in Sudan. Exam anxiety is of recognizable association with academic performance and students' sociodemographic characteristics; thus, we also searched for possible association with students' academic performance and sociodemographic factors. In addition, we went for exploring possible correlation between exam anxiety status and students' level of depression. We found that majority of students have moderate to high test anxiety, while moderates to sever depression was present among more than 50% of the students.These findings are in line with similar results from similar studies [24, 25]. Students' high level of exam anxiety or depression are attributable to many factors: the third year of secondary education in Sudan is the determining year of students' future career path; students perceive this year as very important determinant of their future life opportunities.Other factors of parental and teacher expectations, latent academic and personal disabilities, perceived pressers of community expectations and fear of not meeting self-expectations are also Of remarkable probability in causing such high levels of exam anxiety and depression among students [26-28]. The analysis of the effect of sociodemographic factors as gender, age, parental education and other factors of academic performance and previous exam experienceon the exam anxiety and depression levels shows that, male students experience higher anxiety level than females. These findings are in contrast of studies from similar Arab context and previous one form Sudan that concluded higher anxiety among females' under graduate students [17]. It is logically acceptable to think of level of exam preparedness as a predicting factor for students' level of exam anxiety. students who are more studious and informed will find it easier for them to deal with the exam question with less stress than those who are not. Many studies and academic platforms inrelevant contexts have promoted such theory [29], in an interesting incorporate with our finding in this study that also revealed low academic performance association with higher exam anxiety and depression.In this context, it could be possible that males pay less effort to academic preparation compared to females; the thing which is causing them higher exam anxiety levels, especially that this assumption is strongly supported by the fact that over the last 20 years, female students tends to acquire more position in the top scoring students in Sudan boarding exams. However, the consideration for the social gender role impact in these results should not be ignored [30]. Male students might have higher sense of responsibility and presser to achieve higher because of their social obligations to succeed and find rewarding jobs and working opportunities as the main breadwinners in comparison to females. Given that our study does not provide information about such questions, considering the scope of our research question, further researches that address these important aspect of adolescent's students' psychosocial problems in Sudan are highly needed and strongly recommended.

Again, the same theory might be used to explain the observation of higher exam stress among students with more experience with the exam setting. Students who must set for the exam more than two times are possibly feeling higher anxiety because of their low preparedness and academically devoted efforts, causing them to set for the exam repeatedly. Another factor that perhaps elucidate this, is the suggestion that the more students encounter negative experiences with the exam setting the more anxious they become [31]. Social context in Sudan may lie beneath the observed association about parental level of education and exam anxiety. The finding that maternal education level is a significant positive predictor for students' anxiety status, in contrast to paternal ones, are consistent with many other studies [24, 25]. The explanations for this could be the pattern and amount of parental children interaction and behaviors. Parental Sense of support, bride and rejection to their kids are acknowledged factors that affects children anxiety status. With increased level of education, parents acquiring of these behavioral skills is more likely [32, 33]. Another factor is the highly demanding socioeconomic context in Sudan that requires fathers to work for extended time and in several jobs, and so have less time to spend and dedicate to their children, that is compensated for by mother's role. However, in this study it is difficult to conclude the exact mechanism by which maternal level of education positively affecting student's exam anxiety level, because higher education levels might also mean less time devoted by mother to their children due to business and profession and consecutively less stress and presser carried out by mother on their children. To answer such question, further research focus in paternal role impact in adolescents and student psychological health in Sudan is needed. Our analysis for the correlation between depression and exam anxiety level revealed high correlation figure. Although it is quite proven that depression and anxiety are the fraternal twins of mood disorders, this critical age stage plus exposure to high stress situation of the national board examination make students more psychologically and mentally vulnerable, especially without availing the proper mental and social health means of counselling services, support clinics and experts in their schools. Other factors including the structure of the academic system, assessment methods, school environments, the reciprocal impact of academic performance and lengthy structure of the boarding exam settings should all be considered as influential factors synergizing the exam anxiety to depression [34]. This study has is limited by the homogeneity of study sample that makes it difficult to draw solid conclusions about variables like exam experience and age; small representation of items of variant age and exam experience make the concluded inferences about such variables relatively weak. Another limitation is the usage of self-reported psychological assessment methods that carries high bias of over or under observation of our outcome of interests. However, this study provided novel information about exam anxiety and depression levels among Sudanese students setting for Sudan national secondary boarding exam, as to our knowledge most studies addresses college and university students with no attention devoted to such critical youth group in such critical milestone of their life.

## Conclusion

In this study, we investigated the predictors, correlations and levels of exam anxiety and depression among third year's students sitting for Sudan national secondary boarding exam in Sudan.We found a high level of both exam anxiety and depression that are highly correlated with each other among students .Male gender, exam setting experiences, academic performance and maternal educational level were significant predictors of exam anxietystatus, in contrast to students age and paternal educational level. Further researches are highly needed for more understanding of how these factors affect student anxiety and mental health, and establishment of adolescents' school based mental health and psychological services are highly needed in Sudan.

### What is known about this topic

- High figures of adolescent's mental health problems reported in Sudan;
- Academic stress is possible cause of menta health problems in Sudan;
- Students based factors affect their mental health outcome due to academic stress.

### What this study adds

- High prevalence of morbid exam anxiety levels and severe depression levels among Sudanese students setting for Sudan national board exam;
- Students, gender, maternal level of education, previous exam experience and academic performance levels are risk factor for students' mental health outcome;
- Student's high exam anxiety problems could underline other serious mental health problems.
